# Supplementary material for: Unraveling the Band Structure and Orbital Character of a π‐Conjugated 2D Graphdiyne‐Based Organometallic Network
Source: Small. 2024 Nov 15;21(10):2406533. doi: 10.1002/smll.202406533 (PMC11899486; doi:10.1002/smll.202406533)
Supplement: Supplementary file 1 — Supporting Information [file SMLL-21-2406533-s001.pdf]

NANO · MICRO
small

Supporting Information

for *Small*, DOI 10.1002/smll.202406533

Unraveling the Band Structure and Orbital Character of a π-Conjugated 2D Graphdiyne-Based Organometallic Network

Paolo D'Agosta, Simona Achilli, Francesco Tumino, Alessio Orbelli Biroli, Giovanni Di Santo, Luca Petaccia, Giovanni Onida, Andrea Li Bassi, Jorge Lobo-Checa* and Carlo S. Casari**

Unravelling the Band Structure and Orbital Character of a π-Conjugated 2D Graphdiyne-Based Organometallic Network

Supplementary Information

Paolo D'Agosta[a], Simona Achilli[b,*], Francesco Tumino[a,c], Alessio Orbelli Biroli[d], Giovanni Di Santo[e], Luca Petaccia[e], Giovanni Onida[b], Andrea Li Bassi[a], Jorge Lobo-Checa[f,g,*,†], and Carlo S. Casari[a,*,†]

[a]NanoLab, Department of Energy, Politecnico di Milano, via G. Ponzio 34/3, I-20133 Milano, Italy
[b]Department of Physics "Aldo Pontremoli", Università degli Studi di Milano, Via G. Celoria 16, I-20133 Milano, Italy
[c]Department of Chemistry, Queen's University, 90 Bader Lane, K7L3N6 Kingston, ON, Canada
[d]Department of Chemistry, Università di Pavia, Via Taramelli 12, I-27100 Pavia, Italy
[e]Elettra Sincrotrone Trieste, Strada Statale 14 km 163.5, I-34149 Trieste, Italy
[f]Instituto de Nanociencia y Materiales de Aragón (INMA), CSIC-Universidad de Zaragoza, E-50009 Zaragoza, Spain
[g]Departamento de Física de la Materia Condensada, Universidad de Zaragoza, E-50009 Zaragoza, Spain
[*]Corresponding authors: carlo.casari@polimi.it, jorge.lobo@csic.es, simona.achilli@unimi.it
[†]These authors contributed equally

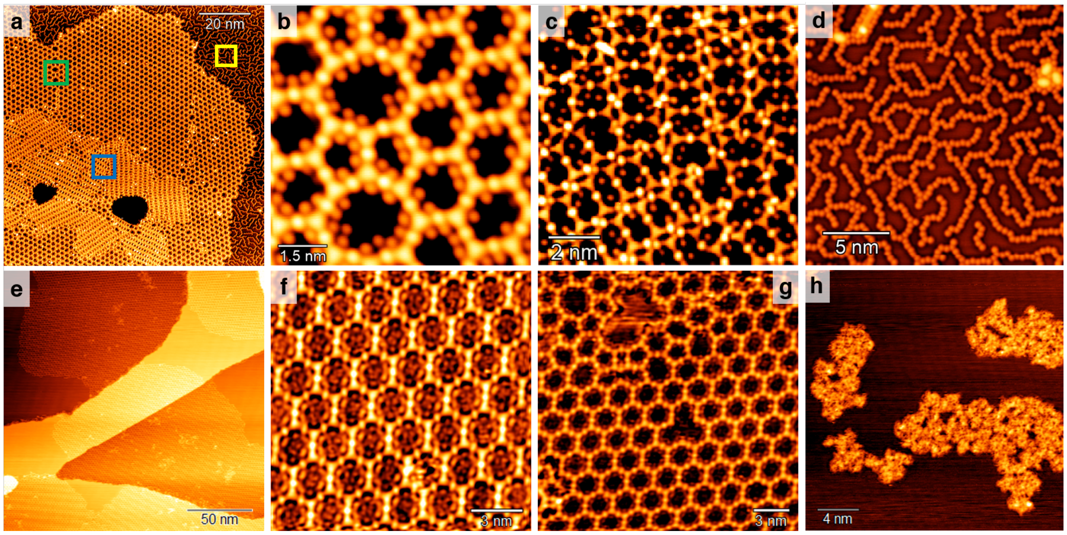

Figure S1: Selection of LT and RT-STM images of 2D organometallic network on Ag(111), highlighting typical defects encountered alongside the mixed honeycomb-kagome 2D network. (a) Large-scale LT-STM image overview of the 2D network after annealing to ∼370 K, showing the coexistence of the porous and compact phase (STM set-point: 1.0 V, 50 pA). (b) Zoom-in at the green square of (a) showing a typical line defect at a grain boundary of coalescing 2D network islands consisting of aligned octagonal and pentagonal pores (STM set-point: −50 mV, 90 pA). (c) Zoom-in at the blue square of (a) displaying the residual compact phase (STM set-point: 70 mV, 100 pA). (d) Zoom-in at the yellow square of (a) showing the atomic arrangement of the cleaved Br atoms on the Ag(111) surface (STM set-point: −1.0 V, 50 pA). (e) Large-scale RT-STM overview image after molecular deposition and annealing on the Ag surface (STM set-point: 700 mV, 400 pA). (f) RT-STM image showing that the 2D network is stable at this temperature (STM set-point: −400 mV, 400 pA). (g) RT-STM image showing the emergence of two different defects with respect to the LT case: a single monomeric vacancy that does not disrupt the surrounding network, and a multiple monomeric vacancy aggregating into a larger network hole that distorts the adjacent pores into rectangles, pentagons, heptagons, or octagons to accommodate the deformation (STM set-point: 600 mV, 400 pA). (h) RT-STM image of the degraded network after annealing to ∼470 K (STM set-point: 700 mV, 400 pA).

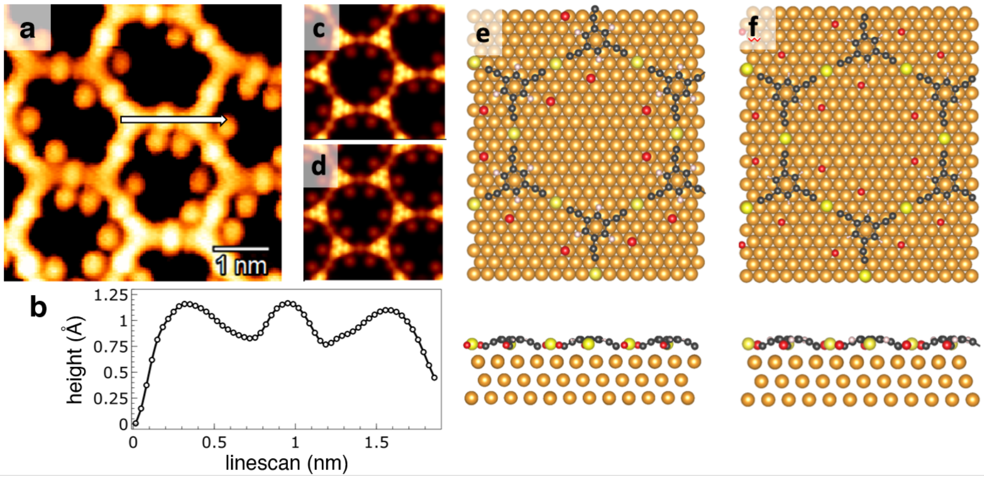

Figure S2: (a) High-resolution LT-STM image (STM set-point: −3.5 V, 800 pA). (b) Average line profile measured along the direction indicated by the white arrow in (a). (c,d) Simulated STM images in the presence of four (c) and six (d) Br atoms in the network, obtained by integrating occupied states in an energy window 0.5 eV wide, at a distance of 2 Å from the surface. (e,f) Simulated atomic structure of the Ag(111)-supported 2D organometallic network in top and side view with four (e) and six (f) Br atoms in each pore. Carbon atoms are in gray, Br atoms in red, Ag adatoms in yellow, and substrate Ag atoms in orange.

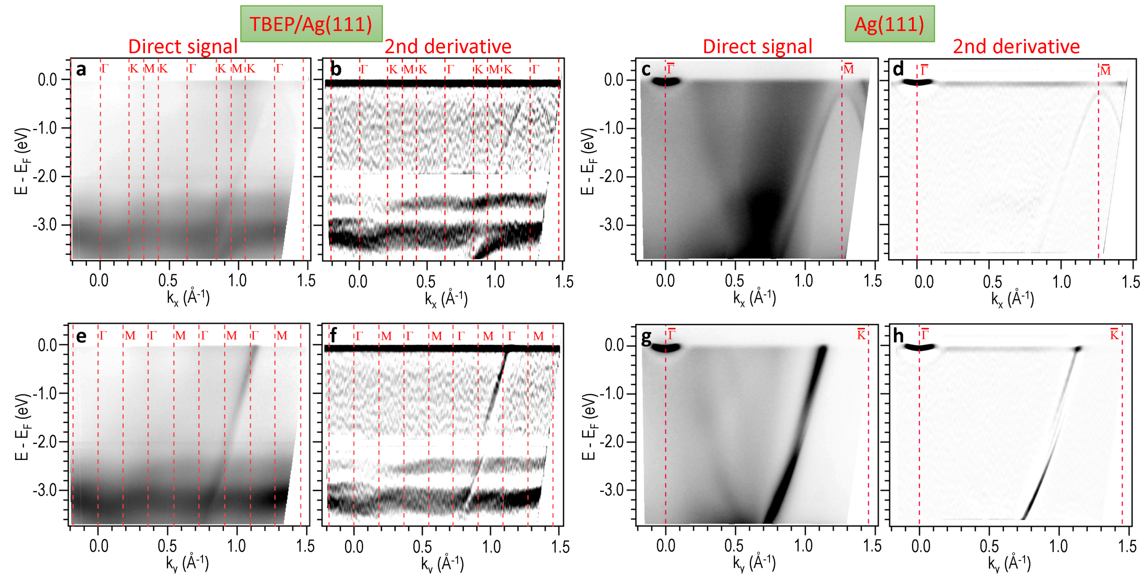

Figure S3: Electronic band structure comparison of the 2D organometallic network versus the Ag(111) substrate along the two high-symmetry directions. The direct photoemission signal, measured with a photon energy of 21 eV, is shown in panels (a), (c), (e), and (g), while the corresponding second derivative in the side panels (b), (d), (f), and (h). The highly dispersive *sp* bands of the substrate at large k_x and k_y values are visible in all panels, whereas the relatively flat band-manifolds at the lowest energy region are only visible in the network's signal (left columns). Contrarily, the Shockley state is only visible at Γ for the pristine substrate, as it is depleted in the 2D network. The color grayscale is linear (the darker, the more intense) and the vertical dashed lines indicate the high-symmetry points (rotated by 30° with respect to one another).

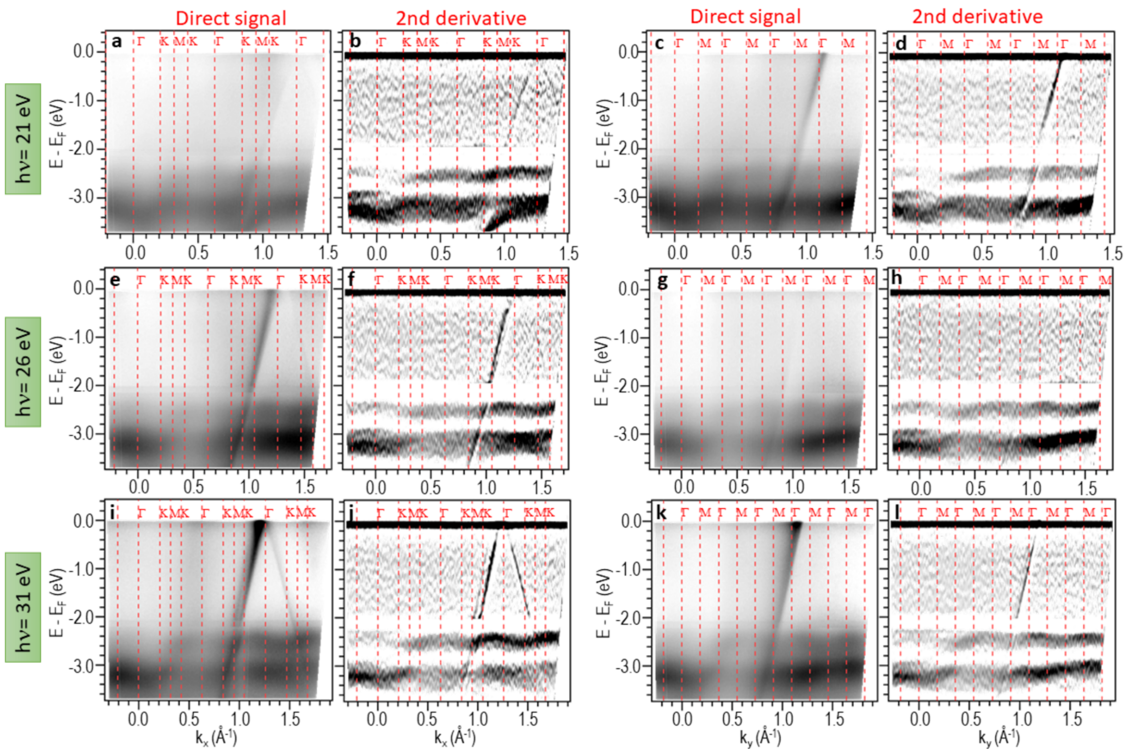

Figure S4: Electronic band structure evolution of the 2D organometallic network as a function of photon energy along the two high-symmetry directions, specifically at 21 eV (a–d), 26 eV (e–h), and 31 eV (i–l). The direct photoemission signal is shown in panels (a), (c), (e), (g), (i), and (k), while the corresponding second derivative in the side panels (b), (d), (f), (h), (j), and (l). The substrate's *sp* bands change their dispersion with the photon energy, whereas the band-manifolds of the network do not, corroborating their 2D origin. The color grayscale is linear (the darker, the more intense) and the vertical dashed lines indicate the high-symmetry points (rotated by 30° with respect to one another).

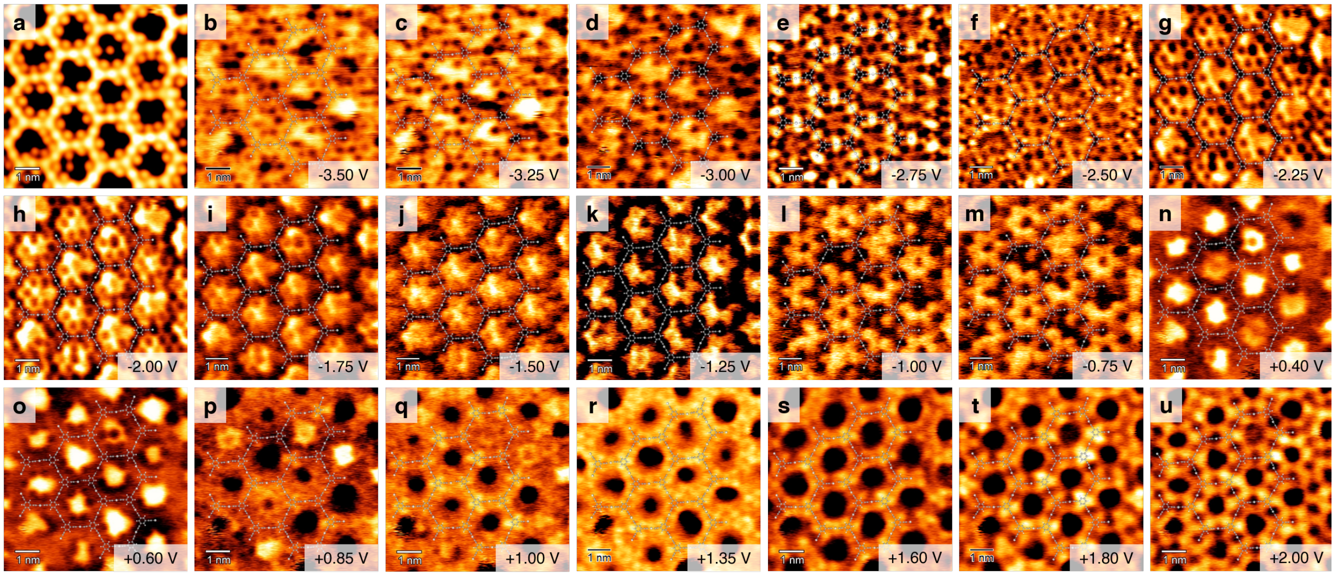

Figure S5: (a) Reference LT-STM image (STM set-point: −2.0 V, 300 pA). (b−u) LT *dI/dV* maps taken at different set-point biases with V_{RMS} = 10.0 mV and f_{osc} = 817.3 Hz. STS set-points: (b) −3.50 V, 450 pA; (c) −3.25 V, 450 pA; (d) −3.00 V, 400 pA; (e) −2.75 V, 400 pA; (f) −2.50 V, 350 pA; (g) −2.25 V, 350 pA; (h) −2.00 V, 300 pA; (i) −1.75 V, 300 pA; (j) −1.50 V, 250 pA; (k) −1.25 V, 250 pA; (l) −1.00 V, 200 pA; (m) −0.75 V, 200 pA; (n) +0.40 V, 100 pA; (o) +0.60 V, 100 pA; (p) +0.85 V, 120 pA; (q) +1.00 V, 120 pA; (r) +1.35 V, 150 pA; (s) +1.60 V, 150 pA; (t) +1.80 V, 200 pA; (u) +2.00 V, 200 pA.

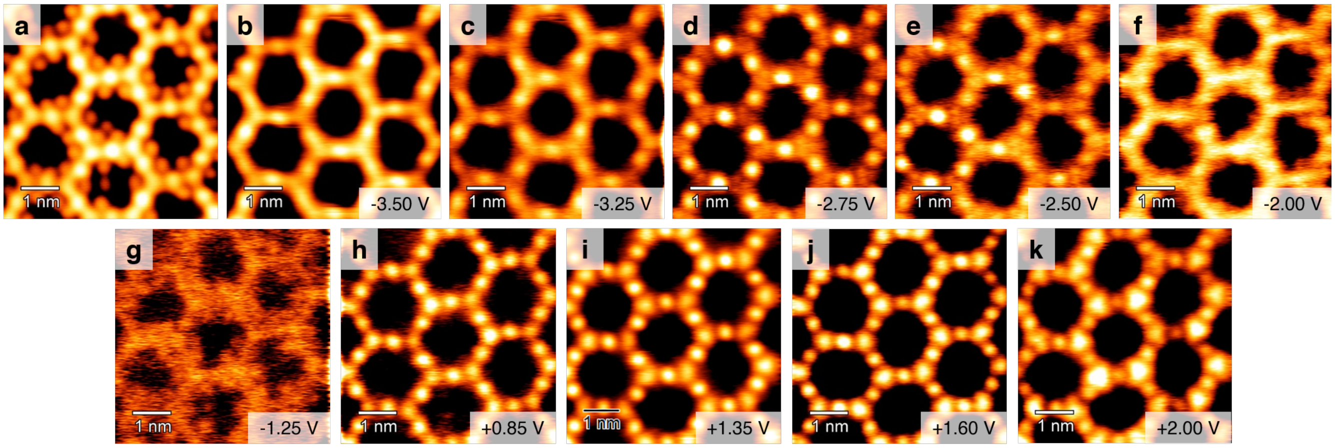

Figure S6: (a) Reference (constant-current) LT-STM image (STM set-point: −100 mV, 50 pA). (b−k) Constant-height LT-STM images taken at different set-point biases. STM set-points: (b) −3.50 V; (c) −3.25 V; (d) −2.75 V; (e) −2.50 V; (f) −2.00 V; (g) −1.25 V; (h) +0.85 V; (i) +1.35 V; (j) +1.60 V; (k) +2.00 V.

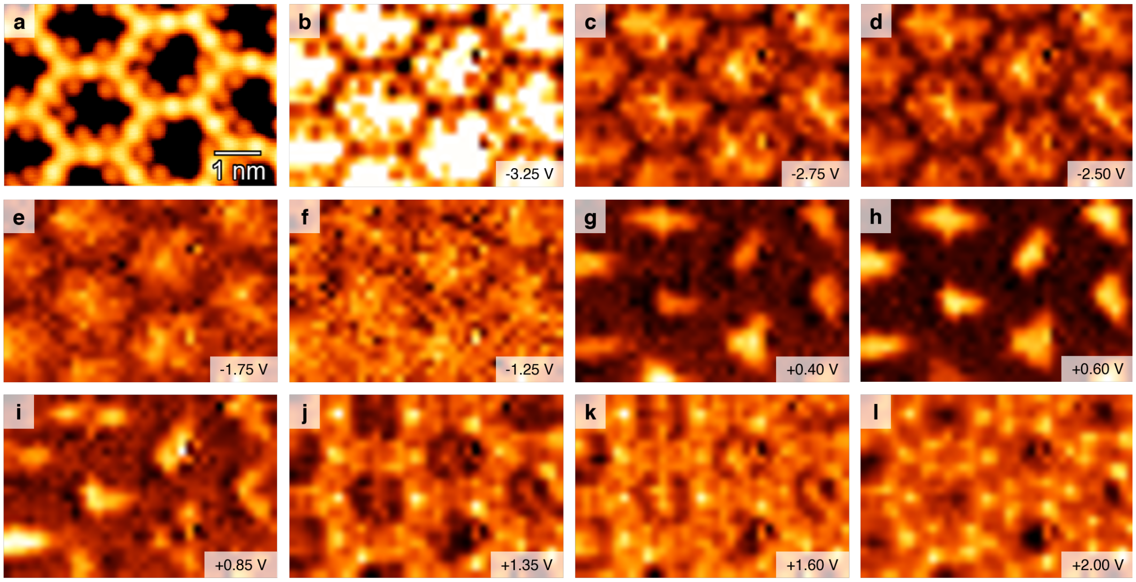

Figure S7: LT-STS maps of a 36×24 pixel grid collected with $V_{\text{RMS}} = 10.0$ mV and $f_{\text{osc}} = 817.3$ Hz. A STS curve is associated with each pixel, and each map is an energy slice at a specific bias voltage. (a) Reference LT-STM image (STM set-point: -500 mV, 14 pA). STS set-points: (b) -3.50 V; (c) -2.75 V; (d) -2.50 V; (e) -1.75 V; (f) -1.25 V; (g) $+0.40$ V; (h) $+0.60$ V; (i) $+0.85$ V; (j) $+1.35$ V; (k) $+1.60$ V; (l) $+2.00$ V.

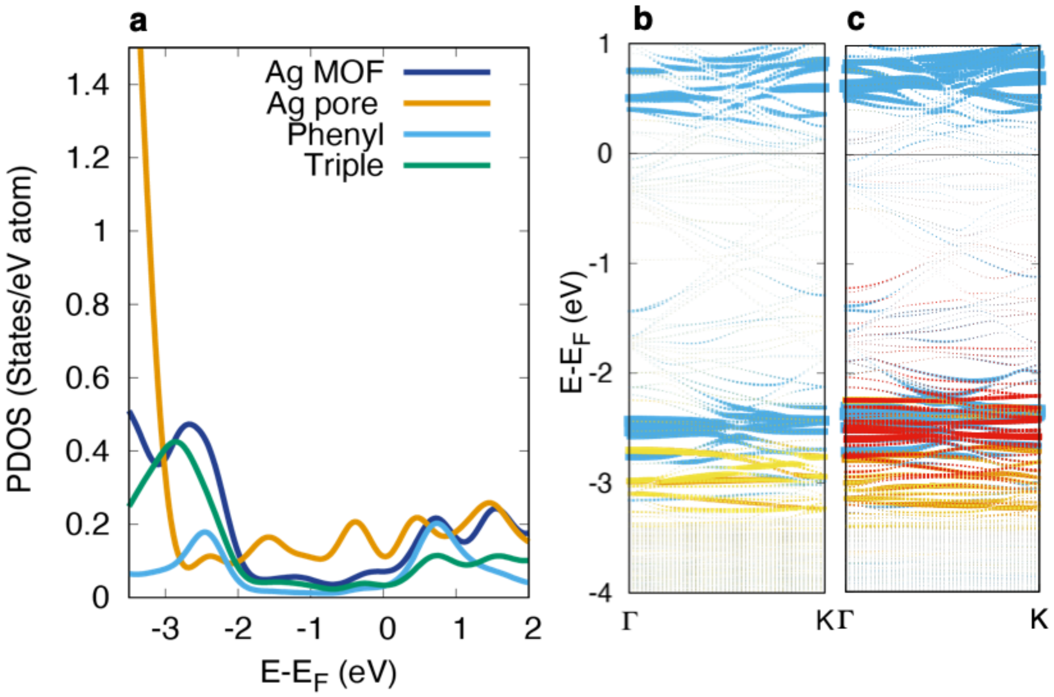

Figure S8: Theoretical electronic properties of the 2D organometallic network on Ag(111). (a) Density of states at the Γ point projected on different groups of atoms. Band dispersion along the high-symmetry path of 2D network without (b) and with (c) Br adatoms in the pores. Carbon bands are reported in blue (p_z), and orange/yellow ($p_{x,y}$). Red bands correspond to states mainly localized on Br atoms.

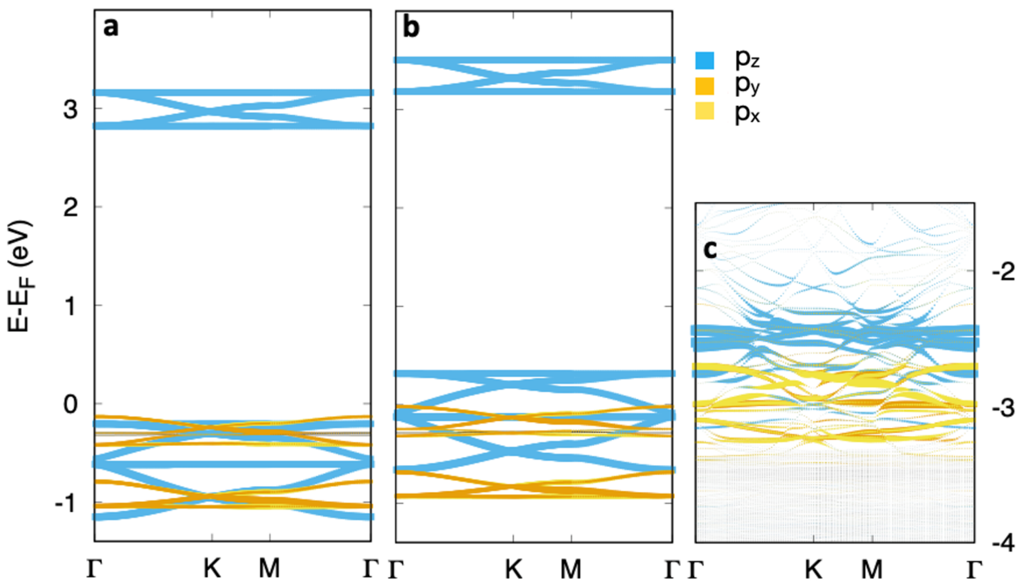

Figure S9: Majority (a) and minority (b) spin band structure of the free-standing 2D organometallic network along high-symmetry paths of the Brillouin zone. (c) Band structure of the Ag(111)-supported 2D network for comparison. Cyan and yellow/orange lines correspond to different p bands, respectively p_z and $p_{x,y}$.

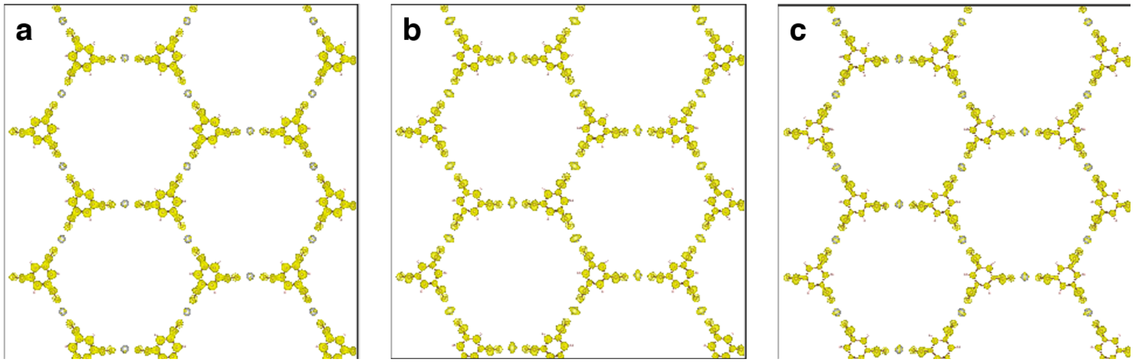

Figure S10: Simulated LDOS of the Ag(111)-supported 2D network at (a) +1.0 eV, (b) +1.3 eV, and (c) +1.5 eV.

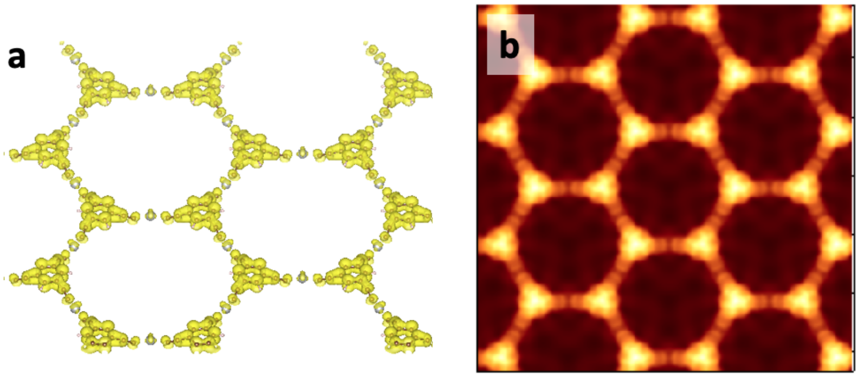

Figure S11: Simulated LDOS (a) and dI/dV map (b) of the Ag(111)-supported 2D network at +2.0 eV from the Fermi level.
